# Retraction Note: Healthcare workers perceptions of patient safety culture in selected Ghanaian regional hospitals: a qualitative study

**DOI:** 10.1186/s40359-026-05178-x

**Published:** 2026-07-23

**Authors:** Demuyakor Isaac, Yuanheng Li, Yushu Wang, Deyou Jiang, Chenggang Liu, Chao Fan, Michael Boah, Yuzhuo Xie, Mingxue Ma, Linghan Shan, Lei Gao, Mingli Jiao

**Affiliations:** 1 Harbin Medical University, Harbin, Heilongjiang China; 2 Northeastern University, Shenyang, Liaoning China; 3 Heilongjiang University of Chinese Medicine, Harbin, Heilongjiang China; 4 Third Affiliated Hospital of Harbin Medical University, Harbin, Heilongjiang China; 5 School of Public health University for Development studies, Tamale, Ghana

**Retraction Note: BMC Psychology 12, 272 (2024)**

**https://doi.org/10.1186/s40359-024-01628-6**

The Editor has retracted this article after concerns about the peer review process prompted post-publication peer review. The review found issues with methodology such as non-transparent recruitment, unclear selection criteria and interview questions - as well as issues with the results such as weak substantiation for the claims made in the article. The authors provided an explanation which was not found to be sufficient.

The authors did not state whether they agree to this retraction.

